# Superbug vs. Superphage A Review of *The Perfect Predator* and an Interview with Dr. Chip Schooley

**DOI:** 10.4269/ajtmh.19-0770

**Published:** 2019-12-02

**Authors:** Claire Panosian Dunavan

**Affiliations:** University of California; Los Angeles, California; E-mail: cpanosian@mednet.ucla.edu

This story reads like science fiction, but it’s not. If you didn’t know that multidrug-resistant bacteria will kill someone every 3 seconds by 2050, you’re not alone. Tom and I were blindsided when he went from being a supremely hardy guy to one who was fighting for his life against the deadliest superbug on the planet. For me, as an infectious disease epidemiologist, my professional and personal lives collided, and I took it personally.

This book isn't just our story; it’s a cautionary tale about the world we now live in, a world on the verge of a post-antibiotic era where a small scrape or a routine surgery could end up being fatal. Find out how we enlisted the help of strangers around the globe to resurrect a hundred-year-old forgotten cure that now holds promise as a potential solution to combat the global superbug crisis.

Steffanie Strathdee, Ph.D., 2019

Every year, a chill wind blows colder, signaling that a stunning twentieth century success could someday bring “antibiotic winter.” And even if we believe science will check the global threat, nothing focuses the mind like a harrowing story. One man’s battle with *Acinetobacter baumannii* presents with a final, surprising twist. His 11th-hour rescue was so dramatic that a famous Hollywood person has now optioned his story for a major motion picture.

So much for my tease for *The Perfect Predator—A Scientist’s Race to Save Her Husband from a Deadly Superbug*. Has any previous book about bacteriology inflamed a similar response? Well, yes. Following its 1931 release, the movie based on Sinclair Lewis’s *Arrowsmith* earned four Academy Award nominations, including Best Picture.

In Lewis’s story, however, an antiserum halted a firestorm of plague. For Dr. Tom Patterson, salvation equaled multiple infusions of bacteriophage specifically crafted to kill his resistant, promiscuous bug.† No previous human had ever received this novel therapy, much less a patient who had already spent 100 days facing death.

*The Perfect Predator’s* coauthors could easily be us. Patterson is a professor of psychiatry at the University of California, San Diego (UCSD), whose research has ranged from HIV/AIDS prevention to rehabilitating patients with dementia—Stephanie Strathdee, his epidemiologist wife, is UCSD’s Associate Dean of Global Health Sciences. The two were vacationing in Egypt when Patterson first suffered gallstone pancreatitis, a condition in which gut bacteria sometimes breach the biliary tree. Roughly a week later, after he was flown by emergency transport from a clinic in Luxor to a university hospital in Germany, a football-sized pseudocyst in Patterson’s pancreas grew a strain of *A. baumannii* that was nearly impervious to treatment. After Patterson was once again med-evac’ed to Thornton Hospital at UCSD, his stowaways lost their final shreds of sensitivity.

That’s when Strathdee started scouring the internet and stumbled across reports of “Trojan horse” viruses that killed dangerous bacteria. “Might one or more phages destroy Tom’s deadly invaders?” she wondered. What followed next was a heroic effort to find, test, and purify the sci-fi–like creatures (in the book, Strathdee likens phages to “… tiny alien spaceships with long spindly legs, not unlike the Star Wars Imperial walker Cameron [her son] used to make from Legos”) by a team including Carl Merril, a retired, veteran NIH scientist‡; creators of customized phage at Texas A&M and a navy laboratory in Maryland; and a San Diego start-up called AmpliPhi Biosciences. Taking the lead at UCSD was Strathdee’s long-time colleague Dr. Robert “Chip” Schooley, an infectious diseases specialist whose career in research had already spanned HIV immunology, treatments for herpes, and the development of drugs for hepatitis C. In addition, Schooley has studied the delivery and use of lifesaving antiretrovirals in Africa. Today, he also serves as editor in chief of *Clinical Infectious Diseases* and, with Strathdee, codirects the recently created UCSD center called I-PATH, which stands for Innovative Phage Applications and Therapeutics.

Now for a quick primer for those who are rusty on phage. First discovered in 1917,§ the tiny subunits are ubiquitous, their numbers exceeding all other organisms combined. Bacteriophage not only inhabit seawater, sewage, and all other sites on our planet but also abound in our digestive, respiratory, and reproductive tracts. They act in myriad ways. Their genetic material can remain dormant or unleash potent bacterial toxins (cholera and diphtheria, for example, are two diseases turned lethal by phage). Most importantly, lytic phage like the Ninja fighters flown to UCSD (see following text) can invade bacteria, amplify their viral kin, then burst out and ablate their bacterial sancta, repeating the process ad infinitum*.* The only glitch? The superheroes can also wilt if their quarry deletes genes encoding their attachment. And this, of course, is just one of several clever maneuvers by which bacteria deflect their bellicose foes.

Back to the book which continues to fly off the shelf. Since its publication in early 2019, reviewers have hailed *The Perfect Predator* with accolades such as “a fascinating and terrifying peek into the devastating consequences of antibiotic misuse…” (Scientific American), “[a] gripping and intriguing medical thriller…” (Publishers Weekly starred review), and “[one of the] best health and science books to read this summer” [STAT News]. Add my name to its fans. Having previously worked with Strathdee on a global health committee for the University of California, I knew she was smart, funny, and tenacious. How much? To find out, just head to your closest bookstore, library, or—in the not-too-distant future—neighborhood theater.

Oh, and one more thing: If you’re looking for a gift for a young person currently considering a career in science or medicine, this is the book to buy. What Sinclair Lewis’s *Arrowsmith*, Paul de Kruif’s *Microbe Hunters*, Hans Zinsser’s *Rats*, *Lice*, *and History*, and Robert Desowitz’s *New Guinea Tapeworms and Jewish Grandmothers* did for earlier generations of curious kids, *The Perfect Predator* could do for their twenty-first century counterparts. Thanks to a cowriter named Teresa Barker, the book is fast paced, smart, and hip. Finally, it also features mystic reveries from some dark, stygian cavern in the mind of its ultimate protagonist, the patient himself. And then, miracle of miracles, Patterson comes back to life! No wonder Hollywood instantly inked a deal.

## INTERVIEW WITH DR. CHIP SCHOOLEY¶

**Let’s start at the beginning. What originally drew you to infectious diseases?** During medical school and my early years of training, I was impressed that infectious diseases used therapeutic agents that mostly worked. We had patients who came in acutely ill—we had medicines to give them—and most of the time we reversed their illnesses pretty quickly. This was particularly true of young people with acute infections. For me, the ability to reverse serious pathology rather than to simply palliate was exciting.

Some of my colleagues chose procedural specialties because they wanted to use their hands and get good at a particular thing. I wanted to face new questions and challenges and think on my feet rather than do the same thing over and over.

**During your career, you’ve certainly worked on diseases that initially seemed hopeless. Are you hopeful by nature?** In general, I’m extremely hopeful. After all, the history of mankind has been a series of challenges that initially seemed hopeless. But with time and thought and investment, we’ve gotten from dying of diarrhea from contaminated water pumps in London, for example, to where we are today. That’s because we kept our eye on the ball and tried to overcome challenges.

Remember, what I first liked about infectious diseases was that people got better. But when HIV came along, all of a sudden people weren’t getting better. What helped me to focus on the disease and avoid becoming psychotically depressed because people my own age were dying all around me was a conviction that—if we worked at it—over time, we would make things better.

By then, I was working on antiviral treatments for herpes at a time when some people believed viruses were untreatable. So, when HIV came along, unlike many of my colleagues who focused on bacteria, I was convinced that—once the pathogen was identified—we could go at it directly and reverse the disease. That’s what kept me coming to work every day for the first nine years of the HIV epidemic. That same hope motivated me to help distribute HIV drugs to resource-limited settings such as Africa.

There are a lot of things that can be done if you decide they’re do-able and work with others with complementary skills and overlapping goals.

**Two quick clinical questions. Before Tom Patterson’s illness, did you ever envision using bacteriophage to treat an antibiotic-resistant infection? And, while treating Tom, what was the moment when you knew he would recover?**No, I really hadn’t thought about using bacteriophage therapeutically. And that key moment was on a Saturday night, about 48 hours after we started the intravenous phages, when Tom woke up and recognized his daughter. Over the previous two days, Tom’s need for pressors had begun to decline and his urine output increased, so as far as I could tell, things were moving in the right direction. But when he opened his eyes and smiled at his daughter, I was really elated. He’d been out for so long we weren’t even sure about the status of his brain. It was extremely affirming.

**Please describe your scientific logic in dosing the phage**. Once we decided to use bacteriophage therapy, given Tom’s precarious state, it didn’t make sense to inch up on his dose; it just made sense to go for it because we needed to turn things around quickly. From [experience with] antiretroviral therapy, we also knew that the easiest way to develop resistance is to gradually increase drugs given in subtherapeutic concentrations.

Bacteriophage therapy is virology upside down. We’re giving viral agents that themselves evolved as opposed to treating viruses with the capacity to evolve. If phages are misused, the bacteria at which they are directed rapidly develop resistance.

Another principle of antiviral therapy when targeting RNA viruses is to combine agents whose resistance pathways don’t overlap. As a result, it seemed natural to treat Tom with a highly concentrated “cocktail” of phages. The ones we used were grown in Tom’s own isolate in laboratories at Fort Detrick, Maryland, and Texas A&M. What also made Tom’s treatment different from other phages given over the last 100 years was new purification technology. This allowed us to give a high intravenous dose without exposing Tom to a lot of bacterial endotoxin.

In fact, our first attempt to purify the phages failed—in other words, we not only had lots of phages but also had lots of endotoxin. After a second round of removing bacterial endotoxin, we finally had batches of phages that were safe to give.

**Three years later, is your research mainly focused on phage? If so, what are the biggest challenges and where are your studies headed?** Currently, working on phage is where my greatest interest and energies lie. I’ve always enjoyed doing new things at the same time doing a number of things in parallel. I’m still serving as editor in chief of Clinical Infectious Diseases. I still have an NIH grant with a colleague to develop long-acting antiretroviral drugs. I still have my project in Mozambique while serving as UCSD’s interim faculty leader of Global Education and Engagement. But what intrigues me most and could have the largest impact down the road is bacteriophage therapy.

So far, the biggest bottleneck is finding phages that can be purified in the same way we purified Tom’s. There are only a few laboratories in the world that can do that, and they have limited capacity. The companies are all focused on clinical trials and don’t have the bandwidth to prepare individual phages for individual patients. The biggest challenge has been to manufacture phages to GMP-like standards to administer them to patients who need them.

[As for I-PATH,] right now we’re working with the Division of Microbiology and Infectious Diseases at NIAID to launch a study using phages in clinically stable patients with cystic fibrosis who chronically shed *Pseudomonas.* A colleague would like to treat patients with infected, implanted prosthetic devices. We’ve also considered treating patients with complex urinary tract infections. So, we’re still weighing options at the same time our number one priority is to conduct the same kind of dosing studies one would do with any new antibiotic. That means, understanding the best dosing strategies in terms of levels, frequency of dosing, and pharmacokinetics.

**Thinking back to your early days in infectious diseases, did you ever imagine our current state of antimicrobial resistance?** Not in the beginning because every couple of years there’d be a new class of antibiotic or we’d see a meaningful modification of an existing drug. As we’ve gotten more asymptotic, the modifications have had less impact. Plus, over time, we created an ecosystem where the microbes kept learning although we did not. Today, even if we stopped using antibiotics in all the hospitals in the world, the antibiotics currently in the environment would continue to select more drug-resistant pathogens.

The clock isn’t going to stop no matter what we do. We definitely need antibiotic stewardship—we definitely need to stop squandering antibiotics in animal feed just to sell animals faster—but none of that is going to stop the changes set in motion by 50 years of antibiotic abuse.

**How can the pharmaceutical industry participate more productively in developing new antimicrobial products, including phage?** Well, there are a number of lessons from HIV therapeutics we can apply. The biggest disadvantage of phage or new antimicrobials in general compared with treatments for diseases such as HIV is that you only use them for a short period of time; therefore, amortization in terms of recovering R&D costs is much more complicated. That’s also why hepatitis C therapeutics are much more expensive per pill than HIV therapeutics despite being made in virtually the same way in the same plants.

At the moment, relatively few fixed cocktails might treat most patients with multidrug *Staph* or *Pseudomonas*. And those phages are already in hand—they just need to be engineered to scale and combined in unique combinations that have intellectual property protection. It doesn’t take a genius to figure that out. But right now there aren’t many in pharma who seem to have figured this out.

I’ve heard a number of people from pharma say: “Unless the products are synthetic, we can’t make money from them.” I translate that to: “We can’t do this sustainably.” So, yes, we do need enough cash in the system to have sustainable development by commercial entities. You just can’t use a bunch of academic laboratories to bring phages to scale.

**Further thoughts about the future of phage?** We’ve been fooling around with antibiotics for 80 years or so, and most of them started with small molecules used by microorganisms to kill each other. In some ways, we’re doing the same thing with phages, except here the antibiotics are living. But I do think people should be a little bit humble about this. I mean, these phages have been developing their bacteria-killing skills for 300 million years. Some of them are pretty damn good at it.

Frankly, these days I also worry we’re dumbing down physicians with the algorithm-driven dribble being pushed on us. This kind of thing can cause some people to think: “Why do something like use a bacteriophage?” We need to make sure that physicians making policy and taking care of patients continue to be curious and incorporate new information rather than waiting for guidelines and consensus panels to tell them what to do.

Finally, I believe people everywhere need to feel comfortable demanding the same quality of medicine. If there is one thing that is global and universal with respect to infections, it’s that we all suffer from the same pathogens with the exception of certain exotic organisms. In an ideal world, there would be no delay between new agents becoming available here and overseas. So, we need new economic models for that to happen—we also need educational models to assure that it’s done intelligently—and we need to empower individual physicians and people running hospitals so they clamor for new products to help their patients.

**Any final comments about *The Perfect Predator*?** Steffanie, whose husband was treated with phage, has had an important impact in bringing Tom’s story to the planet and reinvigorating the field in a way that could really affect how phage are viewed, both scientifically and otherwise. That’s not a role I should be playing because, if I do, I lose my credibility as a scientist. On the other hand, as a physician, I had a great experience with Tom Patterson and I’d love to see that happen again and again for other patients. I think it was the phages [that saved him], but we still need rigorous clinical trials to sort that out. That’s where I have come from all my life. I was very enthusiastic about antiretroviral therapy, but it was clinical trials rather than patient testimonials that brought us where we are today. I feel the same way about phage.

Steffanie’s story is also important because it teaches people not to buy into fatalism. These days, too many patients and families have that tossed in front of them because others aren’t thinking creatively. Steffanie was an incredible advocate for Tom and never gave up. Sharing that has meant a lot to her, and it has also meant a lot to people who have faced similar situations.

We actually have a pretty good system, and we should use it more often. Steffanie didn’t have to chain herself to the doors of the FDA for phages to be used in Tom. She didn’t have to throw herself down in the ICU and scream and yell. She didn’t have to go on Facebook and Twitter and say: “You need to GoFundMe so we can keep my husband alive.” She had a patient she loved who had insurance and access to health care as well as physicians who were open to new approaches. And when those new approaches came along, three institutions—and by that, I mean UCSD, Texas A&M, and the U.S. Navy laboratory at Fort Detrick—stuck out their necks along with regulators who said: “That’s what we’re here for. Risks and benefits should be weighed, but we’re willing to let you move forward.”

This story reaffirms that medicine in America is not dead. Medicine in America has a lot of hope. We need to continue to believe that and push forward to make life better for everybody.

**Chip, one last question: You know you’ll be a character in this film. Have you thought about who might play you?**

[Schooley laughs] I’m thinking Jack Black. But seriously, if I’m simply selling popcorn, I’ll be fine with it.

## Footnotes

† *Acinetobacter baumannii* is an increasingly important nosocomial pathogen that readily acquires antibiotic resistance by several means including transposons that facilitate horizontal gene transfers from other bacteria.

‡ Merril spent most of his long career studying bacteriophage and eventually became the chief of biochemical genetics at an NIH laboratory. He was always interested in bacteriophages as human therapeutics.

§ Although another scientist reported antibacterial activity by a “filterable agent” in India’s Ganges and Yamuna rivers as early as 1896, Félix d’Hérelle, a French-Canadian microbiologist, is credited with the discovery and earliest use of bacteriophage as a medical therapeutic. In 1917, d’Hérelle reported an “invisible, antagonistic microbe of the dysentery bacillus” and immediately understood that a virus was parasitizing the bacterium.

¶ This interview was conducted by phone on September 10, 2019 and was later edited for clarity and length.
